# Acquisition and maintenance of pluripotency are influenced by fibroblast growth factor, leukemia inhibitory factor, and 2i in bovine-induced pluripotent stem cells

**DOI:** 10.3389/fcell.2022.938709

**Published:** 2022-09-14

**Authors:** Ramon Cesar Botigelli, Naira Carolina Godoy Pieri, Brendon William Bessi, Lucas Simões Machado, Alessandra Bridi, Aline Fernanda de Souza, Kaiana Recchia, Paulo Fantinato Neto, Pablo Juan Ross, Fabiana Fernandes Bressan, Marcelo Fábio Gouveia Nogueira

**Affiliations:** ^1^ Multiuser Facility (FitoFarmaTec), Department of Pharmacology, Biosciences Institute (IBB), São Paulo State University (UNESP), Botucatu, Brazil; ^2^ Laboratory of Molecular Morphophysiology and Development (LMMD), Department of Veterinary Medicine, Faculty of Animal Science and Food Engineering (FZEA), University of São Paulo (USP), Pirassununga, Brazil; ^3^ Paulista School of Medicine (EPM), Laboratory of Neurobiology, Department of Biochemistry, Federal University of São Paulo (UNIFESP), São Paulo, Brazil; ^4^ Laboratory Biomedical Science, Department of Biomedical Science, Ontario Veterinary College (OVC), University of Guelph, Guelph, ON, Canada; ^5^ Laboratory Department of Animal Science, University of California, Davis, Davis, CA, United States; ^6^ School of Sciences and Languages, Laboratory of Embryonic Micromanipulation, Department of Biological Sciences, São Paulo State University (UNESP), Assis, Brazil

**Keywords:** bovine, induced pluripotent stem cells, embryonic stem cells, cellular reprogramming, pluripotency maintenance

## Abstract

Several opportunities for embryo development, stem cell maintenance, cell fate, and differentiation have emerged using induced pluripotent stem cells (iPSCs). However, the difficulty in comparing bovine iPSCs (biPSCs) with embryonic stem cells (ESCs) was a challenge for many years. Here, we reprogrammed fetal fibroblasts by transient expression of the four transcription factors (Oct4, Sox2, Klf4, and c-Myc, collectively termed “OSKM” factors) and cultured in iPSC medium, supplemented with bFGF, bFGF2i, leukemia inhibitory factor (LIF), or LIF2i, and then compared these biPSC lines with bESC to evaluate the pluripotent state. biPSC lines were generated in all experimental groups. Particularly, reprogrammed cells treated with bFGF were more efficient in promoting the acquisition of pluripotency. However, LIF2i treatment did not promote continuous self-renewal. biPSCs (line 2) labeled with GFP were injected into early embryos (day 4.5) to assess the potential to contribute to chimeric blastocysts. The biPSC lines show a pluripotency state and are differentiated into three embryonic layers. Moreover, biPSCs and bESCs labeled with GFP were able to contribute to chimeric blastocysts. Additionally, biPSCs have shown promising potential for contributing to chimeric blastocysts and for future studies.

## Introduction

In 2006 and 2007, Yamanaka’s group (2006 and 2007) reported that somatic cells could be reprogrammed into pluripotent stem cells (PSCs) by overexpression of a set of four transcription factors (POU5F1, SOX2, KLF4, and c-MYC) (Takahashi et al., 2007; Takahashi and Yamanaka, 2006). These cells named induced pluripotent stem cells (iPSCs) were proven to be self-renewing and could be derived from almost any type of differentiated cell, providing researchers with a valuable source of pluripotent stem cells. The iPSC technology enabled new approaches for stem cell research, including the generation of patient-specific stem cells for regenerative disease modeling and autologous transplants. More recently, the generation of iPSCs from a wide range of species was achieved, including cows (Huang et al., 2011; Heo et al., 2014; Lin et al., 2014), sheep (Li et al., 2011; Liu et al., 2012), horses (Nagy et al., 2011; Whitworth et al., 2014), and pigs (Cheng et al., 2012; Fujishiro et al., 2012; Zhang et al., 2015).

The pluripotency of cells can be classified into two categories, namely, naïve and primed. In rodents, naïve cells are compared to an inner cell mass (ICM) of blastocysts, while primed cells are similar to epiblast cells in the post-implantation embryo (Nichols and Smith, 2009; Hassani et al., 2014; Weinberger et al., 2016). Embryonic stem cells (ESCs), in the naïve state of pluripotency, are dependent of leukemia inhibitory factor (LIF), 2i (MEK/ERK (PD0325901), and GSK-3β (CHIR99021) inhibitors (Nichols and Smith, 2009). Traditional human ESCs are classified as primed as they resemble murine epiblast cells and are dependent of FGF and TGFβ pathways (Hassani et al., 2014). Recently, a “new” state of pluripotency with the capacity to contribute to chimeric embryos, called the intermediate state, was described (Yu et al., 2020; Guo et al., 2021). This intermediate state is dependent of FGF, TGFβ, and WNT pathways. Also, the formative cells present the characteristics of direct *in vitro* primordial germ cell-like induction and chimeric contribution to embryos.

Recently, efficient bESC establishment was described in the MEF feeder (Bogliotti et al., 2018) and feeder-free conditions (Soto et al., 2021). In both reports, cells were isolated using an NBFR base medium with FGF, TGFβ, and WNT pathway modulation. Furthermore, Zhao et al. (2021) reported the derivation of bovine embryonic cells with expanded potential, which resemble a naïve pluripotency state by adding a cocktail of multiple small molecules and cytokines into mTeSR1 media.

The potency of PSCs can be defined using several approaches by identifying molecular markers or functional assays. Also, multiple techniques can be used to identify transcripts, protein expression, and epigenetic profiles. The gold standard functional assay to classify PSCs as naïve is the capacity of the cells to contribute to chimeric embryos (somatic and germline). On the contrary, primed cells do not have this capacity (Nichols and Smith, 2009).

For this, the study presents a comparison of biPSCs derived using either bFGF or LIF in combination or not with 2i and bESCs established in the NBFR medium (Bogliotti et al., 2018; Soto et al., 2021). The characterization of the resultant bovine PSC lines included valuation of pluripotency marker expression by alkaline phosphatase, quantitative RT-PCR, and immunocytochemistry. Also, the potential for our bovine PSC differentiation was evaluated by embryoid body (EB) formation and contribution to blastocysts. The availability of bovine PSCs (iPSCs and ESCs) opens new opportunities for genome editing, production of transgenic animals, genomic selection, and prospects of other reproductive biotechnologies.

## Material and methods

### Cells and media

Mouse embryonic fibroblasts (MEFs), bovine fetal fibroblasts (bFFs), and HEK293t cells were cultured in complete Iscove’s Modified Dulbecco’s Medium (IMDM) [cat. 12200036; Gibco] supplemented with 10% fetal bovine serum (FBS) [cat. SH30071.03, GE Healthcare Life Sciences], 1% GlutaMAX [cat. 35050061, Gibco], 1% MEM Non-Essential Amino Acids Solution [cat. 11140076, Gibco], and 1% penicillin/streptomycin [cat. 15140122, Gibco]. Derived biPSCs were cultured in KnockOut DMEM-F12 [cat. 12660012, Gibco] supplemented with 20% KnockOut Serum Replacement [cat. 10828028, Gibco], 1% GlutaMAX, 1% MEM Non-Essential Amino Acids Solution, 1% penicillin/streptomycin, and 1:1000 β-mercaptoethanol [cat. 21985023, Gibco]. Isolated bESCs were cultured in DMEM-F12 medium [cat. 11320033, Gibco], neurobasal medium [cat. 21103049, Gibco], 1% BSA fatty acid-free [cat. 0219989980, MP Biomedicals], B27 Supplement 50x [cat. 17504044, Gibco], N2 Supplement 100x [cat. 17502048, Gibco], 1% GlutaMAX, 1% MEM Non-Essential Amino Acids Solution, 1% penicillin/streptomycin, 0.1 mM 2-Mercaptoethanol, 20 ng/ml bFGF Peprotech [cat. 100-18B, Peprotech], and 2.5 μM IWR-1 [cat. I0161, Sigma].

### Feeder cells

EmbryoMax^®^ primary mouse embryonic fibroblasts (PMEF-CF, Sigma-Aldrich) were cultured in IMDM medium. Cells were treated with mitomycin C (cat. 47589, Millipore) and used as a feeder monolayer at a concentration of 1 × 10^5^ cells in 6-well dishes. The dishes were treated with 0.1% gelatin before cell seeding.

### Lentivirus production

Lentiviral production was performed as described before (Bressan et al., 2020). Briefly, 5 × 10^6^ HEK-293FT cells were seeded in T75 cm^2^ flasks and maintained in an IMDM with 10% FBS overnight. The STEMCCA vector (12 μg) containing the murine transcription factors OCT4, SOX2, KLF4, and c-MYC (Sommer et al., 2009) and the auxiliary plasmids (1.2 μg TAT, 1.2 μg REV, 1.2 μg Hgpm2, and 2.4 μg VSVG) were transfected using Lipofectamine 3000 reagent (Invitrogen) following the manufacturer’s suggestions. Additionally, the FUGW vector (5 μg) (Lois et al., 2002) and the auxiliary plasmids (1.2 μg pLp1, 1.2 μg pLp2, and 2.4 μg VSVG/pLp) were used for eGFP lentivirus generation used in tracking experiments. The media containing the lentivirus particles were recovered at 24–72 h after transfection. The supernatant was filtered (0.45-µm membrane, cat. SCHVU01RE, Millipore) and distributed media evenly into ultracentrifuge tubes. The ultracentrifuge was functioned at 16,500 RPM, 1:30 h at 4°C (Beckman Coulter, SW28 rotor). The supernatant was removed leaving ∼100 µl per tube to resuspend the lentivirus stock. The lentivirus stock was split into aliquots and stored at - 150°C.

### Bovine FFs and bovine-induced pluripotent stem cell production

Bovine FFs were obtained from 40 to 45 day-old male fetuses and cultured in IMDM medium. For the reprogramming protocol, the isolated fibroblasts were seeded 24 h before lentivirus transduction on 0.1% gelatin-coated wells of a 6-well plate at a density of 2 × 10^4^ cells/well. A fresh medium with lentivirus stock was added to the culture medium, incubated overnight, and replaced by a lentivirus-free medium the following day (transduction was considered day 0). On day 5, the cells were harvested with TrypLE and transferred into an MEF feeder, inactivated by mitomycin-C. MEF feeder layers with reprogrammed cells were cultured in iPSC medium, supplemented with 10 ng/ml bFGF [cat. 100-18B, Peprotech] or ESGRO^®^ Recombinant Mouse LIF Protein [cat. ESG1106—Millipore] associated or not with 2i (1 mM PD0325901 [cat. 444968, Sigma] and 3 mM CHIR99021 [cat. 361571, Sigma]) at 38.5°C, 5% CO_2_, and 5% O_2_ in a humidified atmosphere. Experimental groups were denominated based on their supplementation, such as bFGF, bFGF2i, LIF, and LIF2i. Additionally, biPSCs were frozen once they reached ∼70% confluency in cryopreservation medium consisting of 90% iPSC medium and 10% (vol/vol) DMSO.

### Bovine *in vitro* embryo production

Bovine ovaries were collected from a local abattoir, and cumulus–oocyte complexes (COCs) were aspirated from selected follicles. Follicles 2–6 mm in diameter were aspirated using a 21-gauge needle. COCs were cultured in Tissue Culture Medium 199 (TCM199) [cat. 11150059, Gibco] supplemented with 10% FBS, 0.2 mM pyruvate, 50 µg/ml gentamicin, 0.5 mg/ml FSH, and 5 IU/ml hCG [Vetecor, Hertape Calier] for 22 h. The *in vitro* fertilization was performed with frozen-thawed semen from a single Holstein bull. Capacitated sperms were obtained after Percoll gradient (45% and 90%) separation. IVF was performed in Tyrode/albumin/sodium lactate/sodium pyruvate (IVF-TALP) medium supplemented with 0.6% BSA. Presumptive zygotes were partially denuded after 18 h and cultured in an SOF medium in an incubator with a humidified atmosphere of 5% CO_2_ and 5% O_2_ in air at 38.5°C for 7 days (insemination was considered day 0).

### Bovine embryonic stem cell isolation and culture

Individual whole blastocysts (day 7) were placed in separate wells of a 24-well dish seeded with a monolayer of inactivated MEFs. Cells were cultured in an N2B27 medium incubated at 38.5°C and 5% CO_2_. Outgrowths (after 6–7 days in culture) were dissociated and passaged using TrypLE express and reseeded in the presence of the Rho kinase (ROCK) inhibitor Y-27632 [10 μM, cat. 72302, STEMCELL Technologies] into newly prepared wells containing MEFs and fresh medium. Once established, bESC lines were grown in the same conditions and were passaged every 3–5 days. To increase cell survival in the replate, the ROCK inhibitor was added to the fresh culture medium for 24 h until the next medium change. The medium was changed daily.

### Histochemistry and immunohistochemistry

Alkaline phosphatase activity was detected with an alkaline phosphatase kit (Leukocyte Alkaline Phosphatase kit, [cat. 86R, SIGMA] according to the manufacturer’s instructions. For immunofluorescence staining, cells were fixed with 4% paraformaldehyde (PFA) in DPBS for 10 min, permeabilized with 1% Triton X-100 in Dulbecco’s Phosphate-Buffered Saline (DPBS) for 20 min, blocked for 1 h with 1% BSA and 0.3% Triton X-100 in DPBS, and incubated with primary antibody overnight at 4°C in blocking solution, anti-Sox2 [cat. Ab97959, 1:300; Abcam], anti-Oct4 [cat. sc-8628, 1:300; Santa Cruz Biotechnology], anti-Nanog [cat. AB80892, 1:250, Abcam], anti-Gata6 [cat. Ab175349, 1:250, Abcam] and anti-trimethyl-Histone H3 (Lys27), also known as Anti-H3K27me3 [cat. 07-449, 1:300, Millipore]. The next day, cells were washed with PBS and 0.05% tween 20 and incubated with secondary antibodies (Alexa Fluor 568-conjugated goat anti-rabbit IgG [cat. A-11036, Life Tech] and Alexa Fluor 488-conjugate goat anti-mouse IgG [cat. A-11029, Life Tech]) for 1 h at room temperature. Then, cells were washed thrice for 10 min each; on the second wash, 10 µg/ml of Hoechst 33342 [cat. B2261-25 MG, Sigma] was included for 10 min. Samples were mounted on microscope slides with Prolong Gold Antifade [cat. P36935, Life Tech]. Images were captured using a confocal microscope [TCS-SP5 AOBS; Leica] using laser excitation and emission filters specific for DAPI 358, Alexa 488, and Alexa 568. Digital images were analyzed by evaluating each nucleus's fluorescent intensity using ImageJ-Fiji image processing software (National Institutes of Health, Bethesda, MD, United States). The fluorescent intensity (average mean gray value) of 568 channels was measured by manually outlining each nucleus and adjusting it against the background.

### RNA extraction and reverse transcription

All samples were submitted to total RNA extraction using TRIzol^®^ protocol [cat. 15596018, Invitrogen]. After extraction, total RNA samples were quantified with NanoDrop^®^ (Thermo Scientific). For DNA digestion and reverse transcription, we adjusted the concentration to 1,000 ng RNA per sample. All samples were submitted to DNA digestion using a DNAse I—Amplification Grade^®^ [cat. 18068015, Invitrogen]. For reverse transcription (RT), the High-Capacity cDNA Reverse Transcription kit was utilized [cat. 4368813, Invitrogen].

### Quantitative RT-PCR

Relative quantification of transcribed targets and reference genes was analyzed by qPCR-RT using the PowerUP SybrGreen^®^ PCR Master Mix reagent [cat. A25742, Applied Biosystems] on the QuantStudio 6 (Applied Biosystems). Targets and reference genes were selected by previous reports (Nichols and Smith, 2009; Hassani et al., 2014; Joo et al., 2014; Petkov et al., 2014; Zhang et al., 2015; Acampora et al., 2016; Bogliotti et al., 2018; Bressan et al., 2020; Bessi et al., 2021). RT-qPCR reactions were carried out in a volume of 10 μl containing 100 nM of each primer, 1X Power UP SybrGreen, 2.5 μl H_2_O, and 1 μl template (four-fold diluted cDNA; 12 ng). The primers were designed using the software Primer-BLAST (NCBI) based upon sequences available in GenBank (Table 1). Cycling conditions for amplification were 95°C for 10 min followed by 45 cycles at 95°C for 15 s, 57°C for 20 s, and 60°C for 40 s. Each sample was analyzed in duplicate for each of the genes. Also, ultrapure DNAse and RNAse-free water were used as a negative control of the reaction. For reference genes, ACTB and PPIA were used. The relative gene expression was performed by 2-ΔCt (Livak and Schmittgen, 2001).

**TABLE 1:** General list of primers used to characterize pluripotency and cell differentiation.

Primer	Transcription ID	Forward (5′ → 3′)	Reverse (5′ → 3′)	Product size
ACTB	NM_173979.3	CAG​CAG​ATG​TGG​ATC​AGC​AAG​C	AAC​GCA​GCT​AAC​AGT​CCG​CC	89
PPIA	NM_178320.2	CATACAGGTCCTGGCATC	CACGTGCTTGCCATCCAA	107
NANOG	NM_001025344.1	CTC​AGC​TAC​AAG​CAG​GTG​AAG​A	ACA​CCC​CTG​GTG​GTA​GGA​AT	153
OCT4	NM_174580.2	GCA​AAC​GAT​CAA​GCA​GTG​ACT​AC	GGC​GCC​AGA​GGA​GAG​GAT​ACG	93
SOX2	NM_001105463.2	ATG​GGC​TCG​GTG​GTG​AAG​T	TGG​TAG​TGC​TGG​GAC​ATG​TGA	178
LIFr	NM_001192263.2	TGG​TGG​ACC​GCA​AAA​GAA​TG	AAG​TAC​GGG​ACC​GCT​TTT​CA	228
STELLA	NM_001111109.2	AGT​GAG​CGG​AGG​TAC​AGG​AT	TCG​CAC​TCT​TGA​TCG​AAT​CTC​A	132
OTX2	NM_001193201.1	ACC​CAG​ACA​TCT​TCA​TGC​GG	AAA​TGG​CTG​GGA​CTG​AGG​TG	243
mOSKM		ACG​AGC​CAC​AAG​CTC​ACC​TCT	GGC​ATT​AAA​GCA​GCG​TAT​CC	221
TUBB3	NM_001077127.1	GGA​TAG​ACC​CCA​GTG​GCA​AT	TTG​TGT​GAA​GAA​GCC​TCG​TTG	88
VIM	NM_173969.3	CTC​CTA​CCG​CAG​GAT​GTT​CG	TGG​ATG​TGG​TCA​CGT​AGC​TC	140
PECAM1	NM_174571.3	AAT​CAG​AGC​GTG​GGC​TCA​AA	ATC​CAC​TGG​GGC​TAT​CAC​CT	147
BMP4	NM_001045877.1	AGC​TTC​CAC​CAC​GAA​GAA​CAT	CAC​CTC​GTT​CTC​TGG​GAT​GC	102
AFP	NM_001034262.2	CGG​ACC​TTC​CGA​GCC​ATA​AC	CTC​TTT​CCC​CAT​CCT​GCA​GAC	154
FOXA2	XM_025001047.1	CGAGCCCGAGGGCTACTC	GTA​CGT​GTT​CAT​GCC​GTT​CA	92

### *In vitro* differentiation

Spontaneous differentiation of bovine iPSCs through embryoid body (EB) formation was performed in plates treated with agarose 0.1% and iPSC media without bFGF, bFGF2i, or LIF. After 5 days in suspension culture, EBs were plated on 0.1% gelatin-coated wells after 7 days and grown in complete DMEM. Cells were collected for RT-qPCR analysis.

### Generation of eGFP-positive bovine-induced pluripotent stem cells and embryonic stem cells

Briefly, bovine iPSCs or ESCs were split into new plates (12-well plate), and 50 μl of FUGW lentivirus stock was added to the medium. The medium was changed daily. After 72 h of transduction, bovine PSCs were evaluated using an EVOS™ digital inverted microscope (Life Technologies). eGFP-positive colonies were manually picked and transferred into new feeder MEF plates to obtain eGFP biPSC and eGFP bESC lines.

### Preparation and bovine pluripotent stem cell contribution to chimeric blastocysts

The embryos produced *in vitro* 108 h after fertilization (day 4.5) that clearly presented more than 32 cells were used and defined as an early morula. Bovine eGFP iPSCs or eGFP bESCs were added to an 80 μl drop of TCM-199 medium under mineral oil containing the embryos to be injected. Single eGFP + cells were collected into a 20-μm injection micropipette. Five to eight cells were introduced into the early morula near the perivitelline space. Groups of 30 embryos were manipulated simultaneously, and each session was limited to 30 min. After microinjection, the injected embryos were cultured in a 1:1 (SOFaa: biPSCs or bESCs medium) medium until day 7 of the embryo development in the same conditions previously described. The contribution of eGFP bovine PSCs was evaluated using an EVOS™ digital inverted microscope (Life Technologies). Only blastocysts with eGFP cells near ICM were classified as presenting chimeric contribution.

### Statistical analysis

All data were analyzed using the SAS program version 9.4 (SAS Inst. Inc., Cary, NC, United States). Data were submitted to analyses of normality using the Kolmogorov–Smirnov test. The data that did not meet the statistical premises were submitted to logarithm transformation [Log(X+1)]. The original or transformed data, when necessary, were analyzed using analysis of variance. Data that were collected over multiple time points were analyzed using a repeated-measures test. The treatment and time effects were evaluated by the Tukey–Krammer test. Fluorescence intensity was analyzed by the Kruskal–Wallis nonparametric test, and treatment effect was evaluated by Dunn’s test. Fluorescence intensity was expressed as median ± interquartile interval, and all other variables were expressed as mean ± standard error of the mean. In all statistical analyses, the level of significance was considered at 5%.

## Results

### Generation and initial characterization of bovine-induced pluripotent stem cell production

**FIGURE 1:**
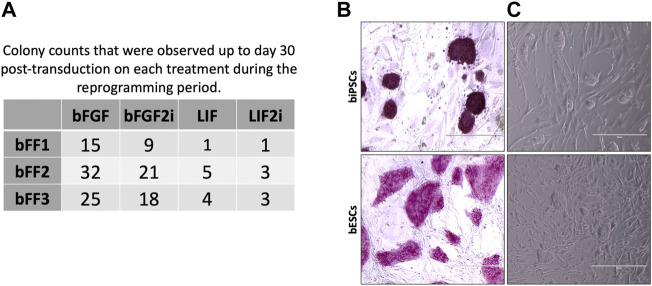
**(A)** Colony counts that were observed up to day 30 post transduction on each treatment during the reprogramming period. **(B)** Representation of alkaline phosphatase detection in biPSCs and bESCs. **(C)** Morphology of biPSC and bESC colonies.

Transduced bFFs (threedifferent lines) were plated on the MEF feeder layers and cultured in bFGF or LIF with or without 2i. Morphology changes on transduced cells were observed after 13 days. Also, we observed variances between the lines on the colony kinetics. All lines when cultured with bFGF during the reprogramming window generated more colonies than other groups (*p* < 0.05) (Figure 1A). Additionally, the LIF2i group did not promote the reprogramming process efficiently (Figure 1A). To follow up on the experiments, we worked with the bFF2 line, and five colonies for each experimental group were expanded by manually selecting colonies (except for the LIF2i group, in which only three colonies were obtained).

Until the third passage, two LIF2i colonies did not grow, suggesting loss of the self-renewal ability and inefficiency of LIF2i to support the reprogramming process in our conditions. Hence, for iPSC line characterization, three clonal lines from each group (bFGF, bFGF2i, and LIF) were used. In passage 3, all biPSC lines were tested for alkaline phosphatase activity, and all were positive. Also, the bESC lines evaluated were AP positive (Figure 1B). The unique LIF2i line did not grow after passage 15, suggesting a loss of the self-renewal ability and inefficiency of LIF combined with 2i to maintain biPSC pluripotency in our conditions.

All biPSCs lines derived from bFGF, bFGF2i, and LIF showed fast proliferation rates (passaged every 4–6 days and reached ∼70% confluency) and high clonogenic capacity and were indicative of self-renewal (were culture until passage 30). These biPSC colonies exhibited dome-shaped morphologies, growing as small, like mESCs (Figure 1C and Additional file 1).

### Gene expression and immunohistochemistry profile on bovine-induced pluripotent stem cells production and bovine embryonic stem cells

**FIGURE 2:**
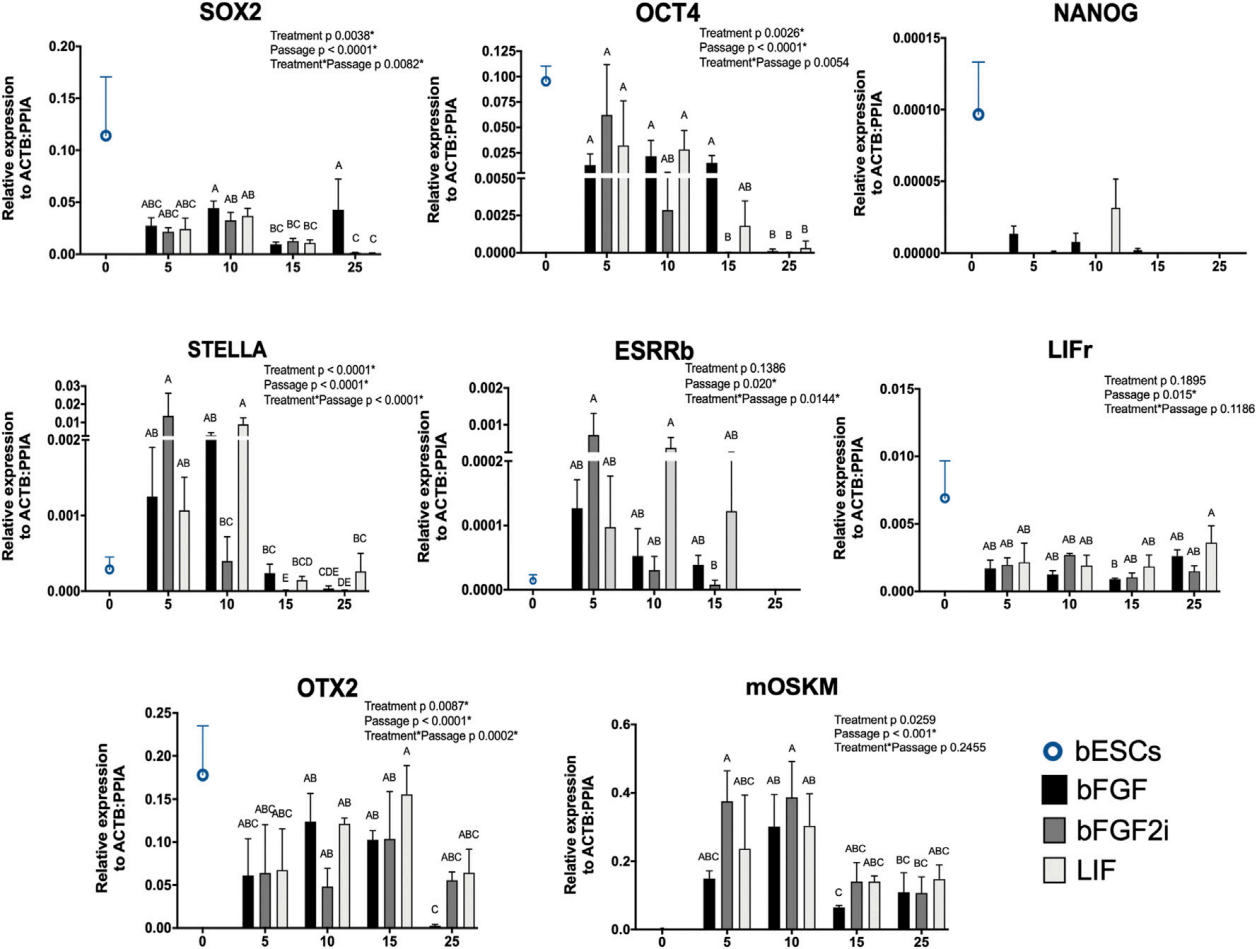
Relative gene expression of SOX2, OCT4, NANOG, ESRRβ, STELLa, LIFr, OTX2 (pluripotent markers), and mOSKM cassette in bovine iPSCs derived on bFGF, bFGF2i, and LIF between passages 5–25 and in bESCs. The X-axis represents the passage of the biPSCs. The Y-axis represents the relative gene expression. Values of *p* to Treatment, Passage, and Treatment Passage were included for each analyzed gene. Bars labeled with different letters are different from each other (*p* < 0.05).

To analyze the reprogramming process, we analyzed all lines at different passages (5, 10, 15, and 25) by RT-qPCR for expression of key endogenous pluripotency factors and the mOSKM cassette. Additionally, we analyzed the bESC gene expression to the same targets as the control of genuine bovine pluripotent cells (Figure 2).

In the first analyzed passage, the biPSCs demonstrated lower levels of SOX2 and NANOG than bESCs. On the other hand, the levels of OCT4 were closer between biPSCs and bESCs. After 10 passages, we observed the levels of both genes (SOX2 and OCT4) decreasing on the biPSC lines (*p* < 0.001, for both genes), except for the bFGF group on SOX2 levels. This group showed the capacity to maintain higher levels of expression even in late passages. Additionally, we observed the expression of NANOG in bESCs and a few samples of biPSCs (bFGF and LIF, passages 5–15). Also, the expression of bESCs was higher than that in biPSC samples.

To better understand the pluripotent profile of the generated biPSCs, we tested our lines for other pluripotent markers, such as ESRRb, STELLA, LIFr, and OTX2. All lines expressed ESRRb, STELLA, LIFr, and OTX2. Interestingly, the levels of biPSCs were higher than those of bESCs to STELLA and ESRRb. Also, the opposite profile was observed to LIFr and OTX2 levels when the expression of bESCs was higher than that of biPSCs. Additionally, the ESRRb, STELLA, and OTX2 levels decreased in relative expression at late passages (*p* 0.0147, *p* < 0.0001, and *p* 0.0002, respectively). Also, the LIFr expression differed between the passages (*p* 0.015), but there was no difference between groups (*p* 0.1895). The exogenous expression of the mOSKM cassette was observed in early and late passages (*p* 5–25). However, mOSKM levels decreased with the passages (*p* < 0.001). We suggest the decreased profile of mOSKM expression can relate to the decreased levels of the endogenous pluripotent genes analyzed here on biPSCs in late passages.

**FIGURE 3:**
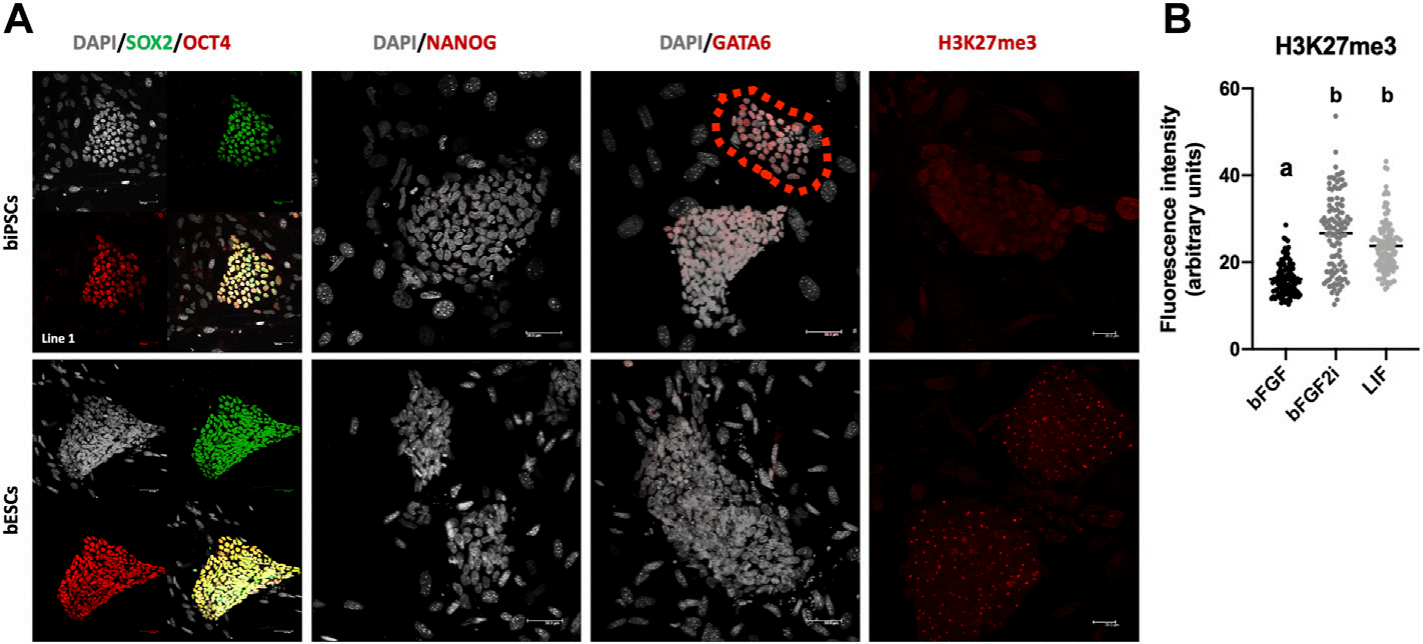
**(A)** Immunofluorescence to SOX2, OCT4, NANOG, GATA6, and H3K27me3 in biPSCs and bESCs. Also, gray cells involve DAPI staining of cells nucleus. Scale bars, 50 μm and 20 μm only to H3K27me3 images. **(B)** Graphical representation of fluorescence intensity to H3K27me3 in biPSCs derived on bFGF, bFGF2i, and LIF. The box plot graphic represented the fluorescence intensity of each analyzed cell, and the data were expressed as median ± interquartile interval.

To characterize the pluripotent protein profile of biPSCs, all lines after passage 25 were tested for pluripotency markers (OCT4, SOX2, and NANOG) and differentiation markers (GATA6) by immunofluorescence. All biPSC lines and bESCs showed pluripotency markers such as OCT4 and SOX2 (Figure 3A). We did not observe differences between the supplementations in biPSCs on SOX2 and OCT4 localization (Additional file 2). We did not observe the presence of NANOG on biPSCs and bESCs, contrasting with the qPCR-RT results (Figure 3A and Additional file 3). The absence of NANOG by immunofluorescence can be correlated with low levels of NANOG expression previously observed.

To identify cells/lines starting the differentiation, we checked for the presence of GATA6. Also, we identified a few colonies expressing GATA6 in one line from the bFGF2i group (Figure 3A and Additional file 4). Immunostaining for the transcriptional silencing-associated marker trimethylation of histone H3 lysine 27 (H3K27me3) was performed (Figures 3A,B and Additional file 5). All lines of biPSCs were positive for H3K27me3; also, we observed a difference in H3K27me3 levels between the groups (Kruskal–Wallis, *p* < 0.0001).

### Differentiation potential of bovine-induced pluripotent stem cells and bovine embryonic stem cells

**FIGURE 4:**
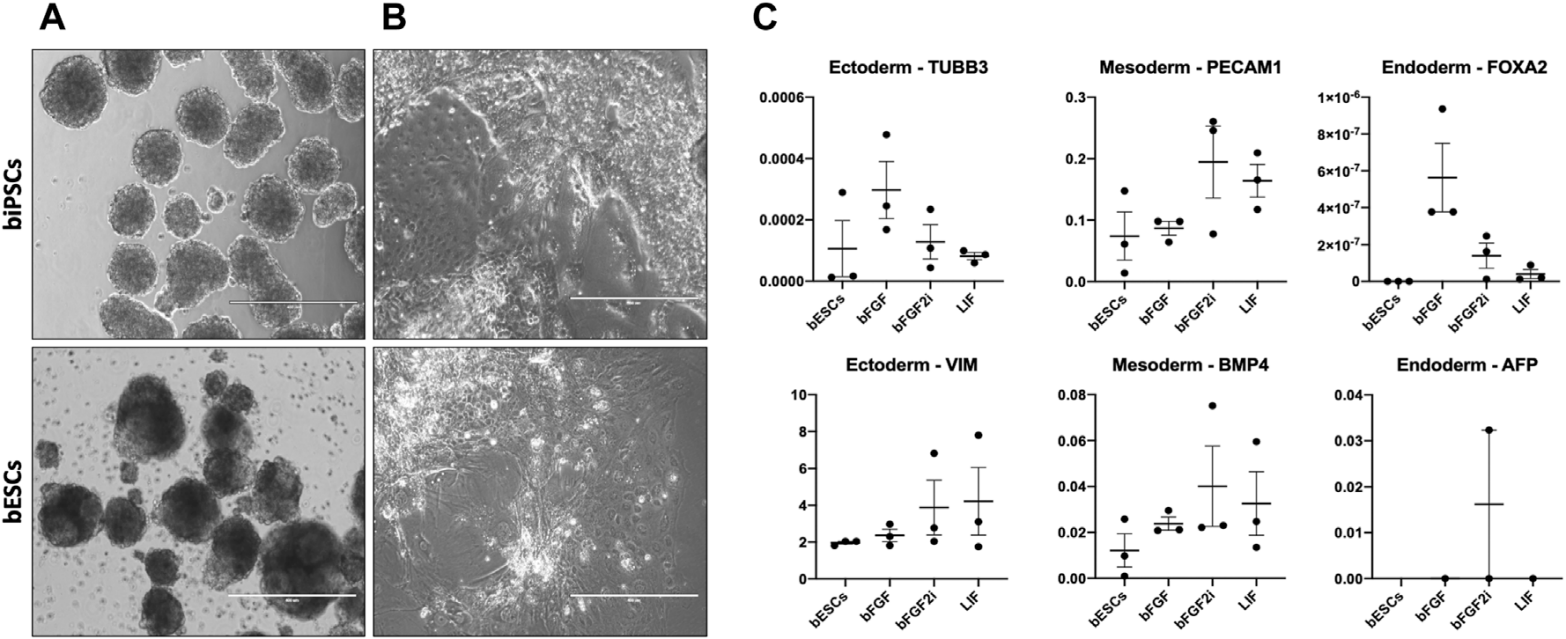
**(A)**, Morphology of embryoid bodies (EBs) produced from biPSCs and bESCs. Scale bars, 400 μm. **(B)** Cells with different morphologies from EBs cultured for 5 days on suspension plus cultured for 7 days in a coated plate with IMDM. **(C)** Relative gene expression of VIM and TUBB3 (ectoderm markers), PECAM1 and BMP4 (mesoderm markers), and AFP and FOXA2 (ectoderm markers) in embryoid bodies. The X-axis represents the group of biPSCs and bESCs. The Y-axis represents the relative gene expression.

iPSCs provide an excellent opportunity for differentiation of any tissue. For this purpose, we cultured the lines on low-adhesion culture dishes for 5 days, allowing them to form EBs to investigate if these cells can differentiate into tissues of the three germ layers (ectoderm, mesoderm, and endoderm). All biPSCs and bESC lines could form spherical structures with defined borders within 24 h of being placed in suspension culture (Figure 4A and Additional file 6). Then, EBs were cultured in coated gelatin dishes to allow further differentiation. After 7 days, different cell morphologies were identified from the EB outgrowth (Figure 4B). Additionally, we evaluated these cells to vimentin (VIM) and tubulin beta 3 (TUBB3) for ectoderm, platelet-endothelial cell adhesion molecule-1 (PECAM-1), and bone morphogenetic protein 4 (BMP4) for mesoderm, and alpha-fetoprotein (AFP) and Forkhead box A2 (FOXA2) for endoderm by RT-qPCR. It was possible to identify ectoderm (VIM), mesoderm (PECAM-1 and BMP4), and endoderm markers (AFP and FOXA2) in most cell lines. Interestingly, two lines from bFGF and all LIF lines lack expression of AFP (Figure 4C).

**FIGURE 5:**
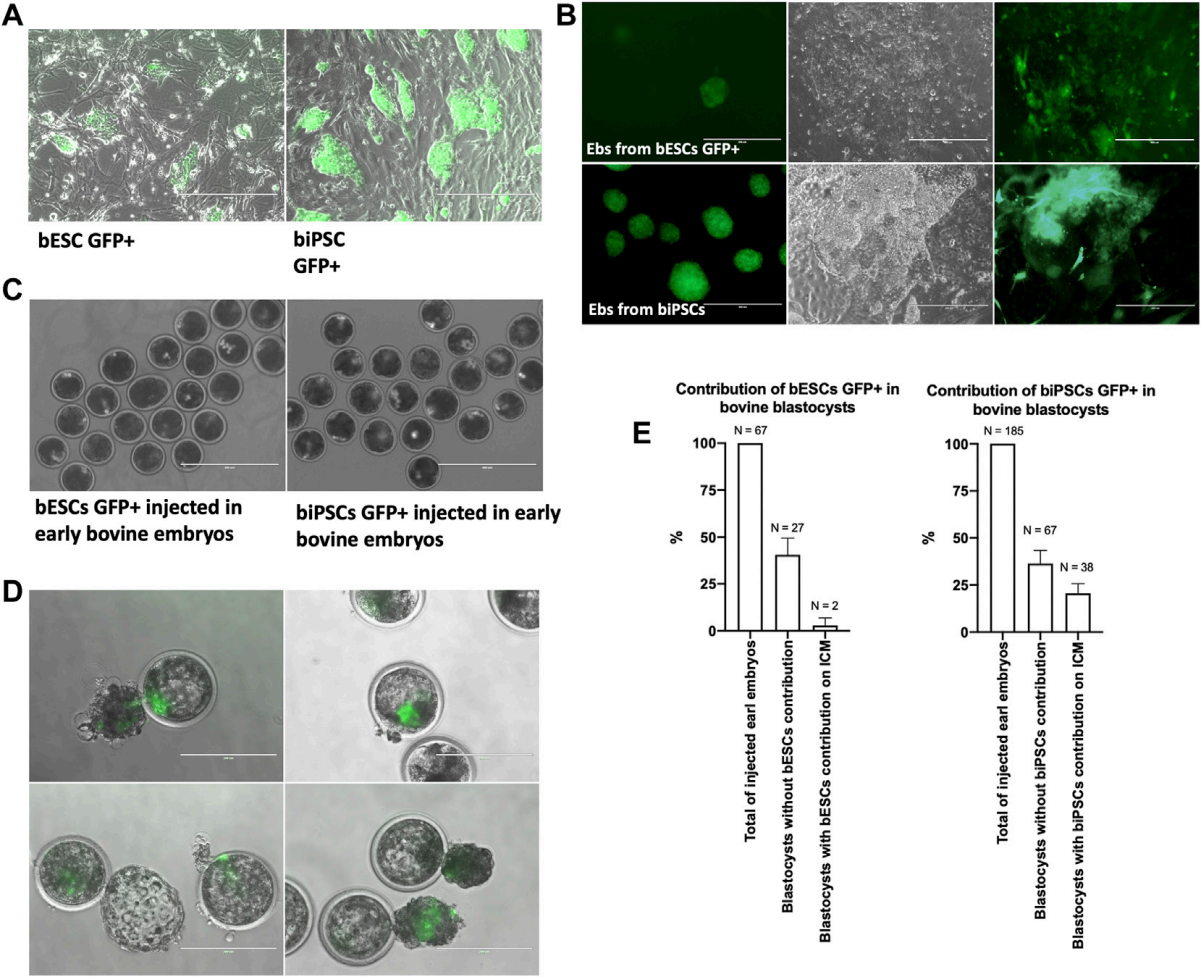
**(A)** Colonies of eGFP bovine iPSCs and ESCS. Scale bars, 400 μm. **(B)** Embryoid bodies (EBs) produced from biPSCs and bESCs GFP+ and cells with different morphologies from EBs GFP + cultured for 7 days in a coated plate with IMDM. **(C)** 5–8 bESCs or biPSCs GFP + injected into each bovine early embryo (day 4.5). Scale bars, 400 μm. **(D)** Bovine blastocysts with the contribution of bovine eGFP iPSCs on the inner cell mass region on day 7 of the embryo development. Scale bars, 200 μm. **(E)** Graphical representation of contribution to eGFP bESCs and eGFP biPSCs on the inner cell mass region on day 7 of the embryo development.

Based on gene expression of SOX2 and the differentiation potential, biPSCs supplemented with bFGF and bESCs lines were injected on morulas to evaluate the potential of these lines in contributing to chimeric blastocysts. First, biPSCs and bESCs GFP+ were produced using FUGW lentiviral (Figure 5A). After that, those cells GFP+ were used to produce EBs and subsequently cultured in IMDM+10% SFB to evaluate differentiated cells expressing GFP (Figure 5B). Then, around 5–8 biPSCs GFP + or bESCs GFP+ were injected into each early embryo (day 4.5) (Figure 5C), and the contribution was evaluated on day 7 of embryo development. We observed only two blastocysts with bESCs GFP + contributing to the ICM, ∼3% of contribution in all injected early embryos (Figure 5E). We observed 38 blastocysts with biPSCs GFP + in the ICM region (Figure 5D), resulting in approximately 20% chimeric blastocysts.

## Discussion

Livestock animals have been used as models for human development and diseases, especially cattle and swine (Navarro et al., 2019; Pieri et al., 2019). The capacity of pluripotent stem cells to differentiate in the three germ lines enables several promising applications, such as cellular therapies, cellular agriculture, and *in vitro* breeding. Many reports described bovine cells submitted to the reprogramming process into iPSCs (Cao et al., 2009; Sommer and Mostoslavsky, 2010; Han et al., 2011; Kawaguchi et al., 2015; Talluri et al., 2015; Zhao et al., 2017; Navarro et al., 2019; Bressan et al., 2020). These different experiments used mostly bovine embryonic or fetal fibroblasts for reprogramming; however, several differences were observed in the reprogramming factors, delivery systems, base medium, and growth factors combined with small molecules or not. Also, none of these studies compared the biPSCs with bovine ESC lines. Here, we present an analysis of biPSCs derived from bFF reprogrammed with mOSKM cultured with bFGF or LIF combined or not with 2i (PD0325901 and CHIR99021). Later, those biPSCs were compared with bESCs.

In our conditions, it was possible to generate biPSC colonies after 15 days of transduction with a polycistronic mOSKM cassette. Also, we observed different kinetics of colony formation using different bFF lines. This result can be correlated with the individuality of each bFF line. Using different reprogramming factors and delivery systems, Heo et al. (2014) (polycistronic human FUW–OSKM/FUW–M2rtTA) and Kawaguchi et al. (2015) (piggyBac transposition of doxycycline-inducible factors, OSK, c-Myc, rtTA, and TagRFP) demonstrated the same reprogramming window (∼14 days) to identify colonies, similar to our results. On the other hand, Huang et al. (2011) used bOSKM and KMOS transcriptional factors (poly-promoter plasmids containing the complete bovine cDNAs for OCT4, SOX2, KLF4, and c-MYC) and identified colonies 8–10 days after the transfection.

Additionally, in the present work and that by Huang et al. (2011), the medium supplemented with LIF2i was not efficient in colony formation, expansion, and self-renewal. We suggest LIF supplementation during the initial reprogramming process can affect the bovine reprogramming. Additionally, we speculate the bovine pluripotent cells do not require LIF supplementation for self-renewal as demonstrated by Soto et al. (2021) and Kinoshita et al. (2021) in bovine ESC feeder-free conditions.

We characterized the pluripotent state of our lines by RT-qPCR and immunostaining. Interestingly, testing the endogenous pluripotent marker expression, we observed in all groups that the relative expression decreases over time after continuous passages (Figure 3). We analyzed these lines with immunostaining after passage 25. At that moment, we detected SOX2 and OCT4 presence. These results corroborate other reports on biPSC-like cells (Sommer and Mostoslavsky, 2010; Huang et al., 2011; Talluri et al., 2015; Zhao et al., 2017). However, we did not detect NANOG expression, post which NANOG protein was not observed. Bogliotti et al. (2018) described a very low expression of NANOG using RNA-seq in bESCs isolated in a customized mTeSR-1 medium supplemented with bFGF and IWR-1. Cao et al. (2009), Heo et al. (2014), and Bressan et al. (2020) demonstrated NANOG mRNA expression and protein presence by immunostaining in bovine iPSCs. Interestingly, the culture condition (MEF feeder vs. feeder-free) can affect the NANOG expression of the bESCs (Soto et al., 2021). Recently, Zhao et al. (2021) and Kinoshita et al. (2021) reported the establishment and continuous expansion of bESCs NANOG positive.

Independent of the reprogramming approach, livestock iPSCs frequently show persistent expression of exogenous transcription factors as a requirement to maintain the undifferentiated state [reviewed by Soto and Ross (2016)]. We observed the same characteristic in our lines based on exogenous vector expression until passage 25. The inability to maintain livestock iPSCs without the reprogramming factor expression has been considered a challenge to report fully reprogrammed cells.

Other important demands involved in iPSCs are the memory of the epigenetic signature from differentiated cells. These changes in the epigenetic profile are necessary to promote the interaction between transcriptional pathways, cellular metabolism, and epigenetic regulation of pluripotency (Hanna et al., 2010; Boland et al., 2014; Wu et al., 2016). H3K27me3 declines in abundance at the 8-cell stage before increasing toward the blastocyst stage of bovine preimplantation embryo development (Ross et al., 2008). In iPSCs, the H3K27me3 reconfiguration is crucial for silencing inappropriate gene expression required for cell lineage specification. Assessing H3K27me3 global levels in our lines, it was possible to identify that bFGF treatment decreased the H3K27me3 levels when compared to the other groups.

Self-renewal is a crucial characteristic to stem cells, ESCs, or iPSCs and essential during replating and the frozen–thawed moments. We observed an interesting connection between frozen–thawed moments. We started frozen–thawed the lines after passage 10 post which all groups showed downregulation of pluripotent marker expression (Figure 3). We did not find explanations or other reports in literature of the same results; however, we suggest that the frozen–thawed process can affect reprogramming.

To test the differentiation capacity of our lines, we submitted all biPSCs to EB assay and chimeric embryo contribution. All tested lines were competent to produce EBs with the expression of ectoderm, mesoderm, and endoderm markers. The absence of AFP endoderm marker expression in a few lines can be due to the timing of the analysis (endoderm tissue needs more time to start the differentiation) or the incapacity of our lines to differentiate into endoderm tissue. On the blastocyst contribution, we observed the contribution of both bovine PSCs (biPSCs and bESCs) to chimeric blastocysts. Additionally, the biPSCs GFP+ when injected in early morulas produced more chimeric blastocysts than bESCs GFP+. These results indicate our biPSCs have a promising potential in differentiation assays (*in vitro* and *in vivo*). bESC contribution to chimeric embryos was reported a few times. Those reports described the ability of the bESCs to contribute to extra-embryonic and placenta tissues (Cibelli et al., 1998; Iwasaki et al., 2000; Saito et al., 2003; Furusawa et al., 2013) and contribute to chimeric fetuses (2021). In our conditions, we observed a cell differentiation potential and low efficiency of blastocyst contribution. This result can indicate not all bESCs have chimera competency (line and culture conditions). For us, especially the supplements such as small molecules and cytokines can influence the pluripotency state of these bESCs (reported here, by Zhao et al. (2021) and Soto et al. (2021)).

Additionally, Kawaguchi et al. (2015) reported for the first time bovine iPSCs with a capacity to contribute to embryonic and extraembryonic tissues. In the present work, we observed the contribution of bovine iPSCs in blastocysts. These results encourage us to continue testing these cells and overcome obstacles to the production of chimeric fetuses in large animals. The present study represents a great advance in the bovine PSC field, especially for open new opportunities and goals to apply the livestock PSCs to chimeric embryo models.

## Conclusion

In conclusion, our results showed that bovine iPSCs could be generated by mOSKM. However, the LIF2i treatment was not viable. Also, the bFGF supplementation was more efficient in promoting the reprogramming of bFFs into biPSCs in our conditions. Additionally, we demonstrated the potential of those lines in different differentiation assays. Future studies need to concentrate their efforts on acquaintance and maintaining pluripotency on iPSCs from livestock once the tool can provide a significant option to study the early development process, cell differentiation, and production of chimeric embryos. The next step to be achieved is the production of adult chimeras and healthy offspring.

## Data Availability

The original contributions presented in the study are included in the article/Supplementary Material; further inquiries can be directed to the corresponding authors.
