# Supplementary figures and images for: Acquisition and maintenance of pluripotency are influenced by fibroblast growth factor, leukemia inhibitory factor, and 2i in bovine-induced pluripotent stem cells
Source: Front Cell Dev Biol. 2022 Sep 14;10:938709. doi: 10.3389/fcell.2022.938709 (PMC9515551; doi:10.3389/fcell.2022.938709)

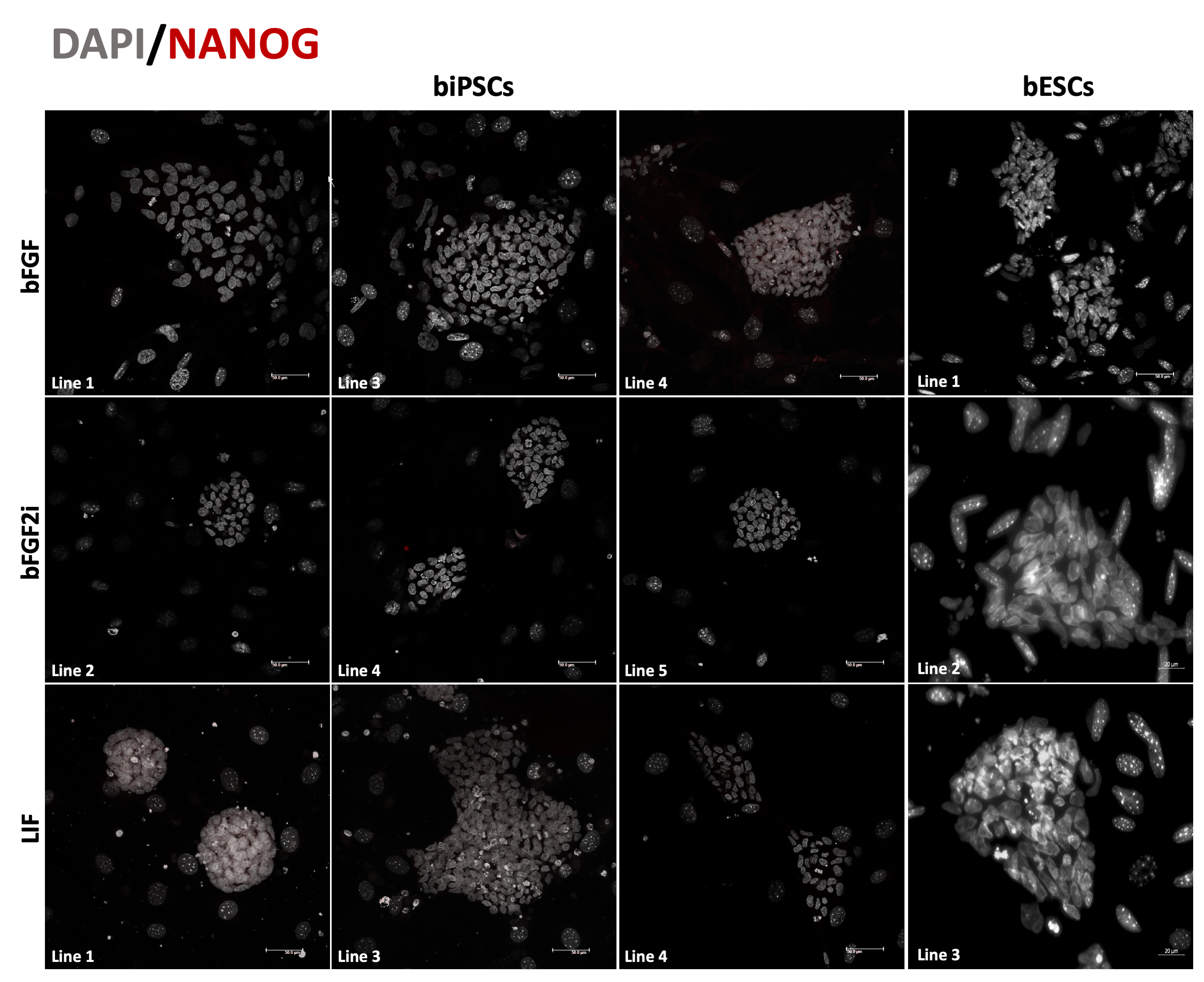

Supplement: Supplementary file 1 [file Image3.TIFF]

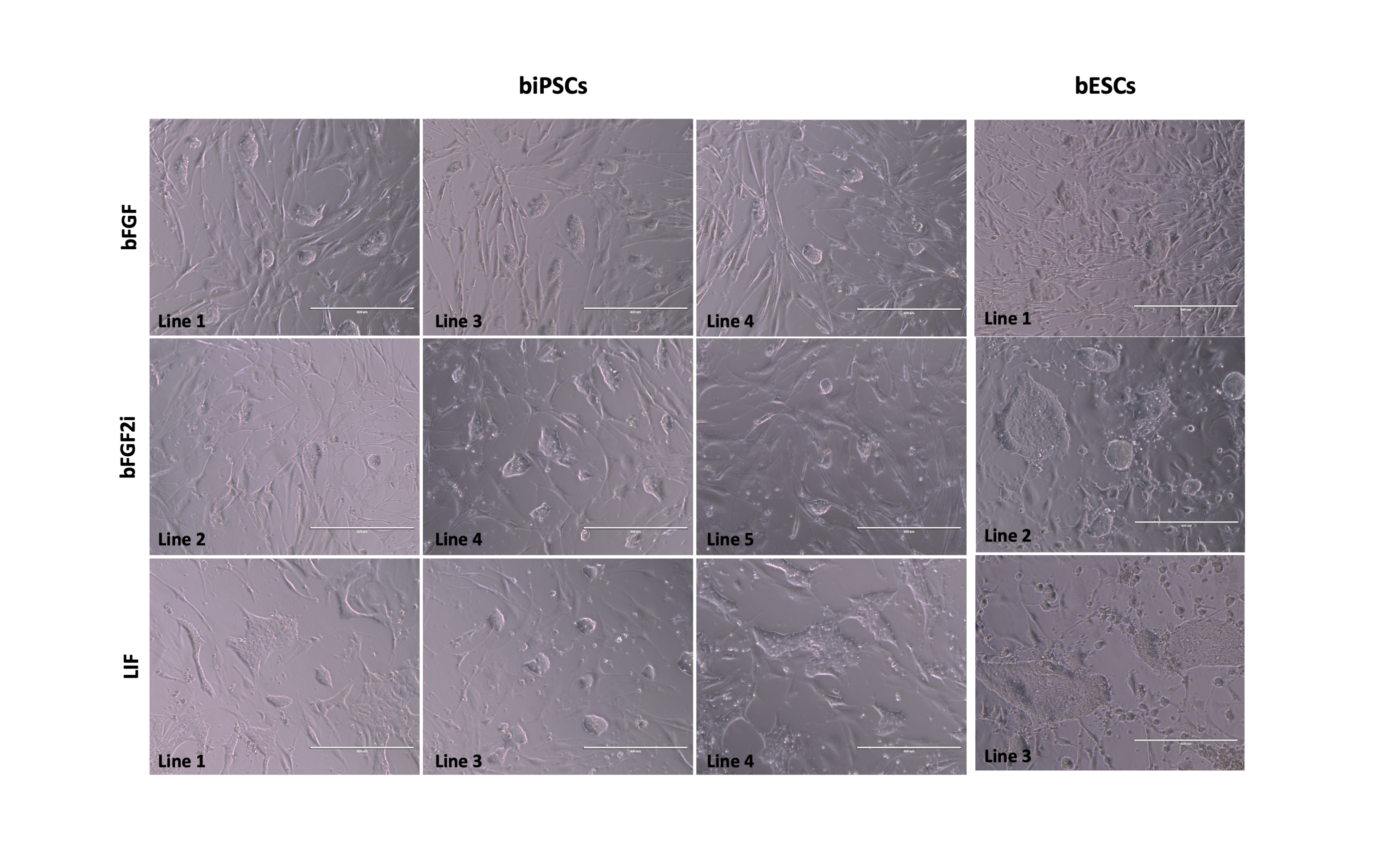

Supplement: Supplementary file 2 [file Image1.TIFF]

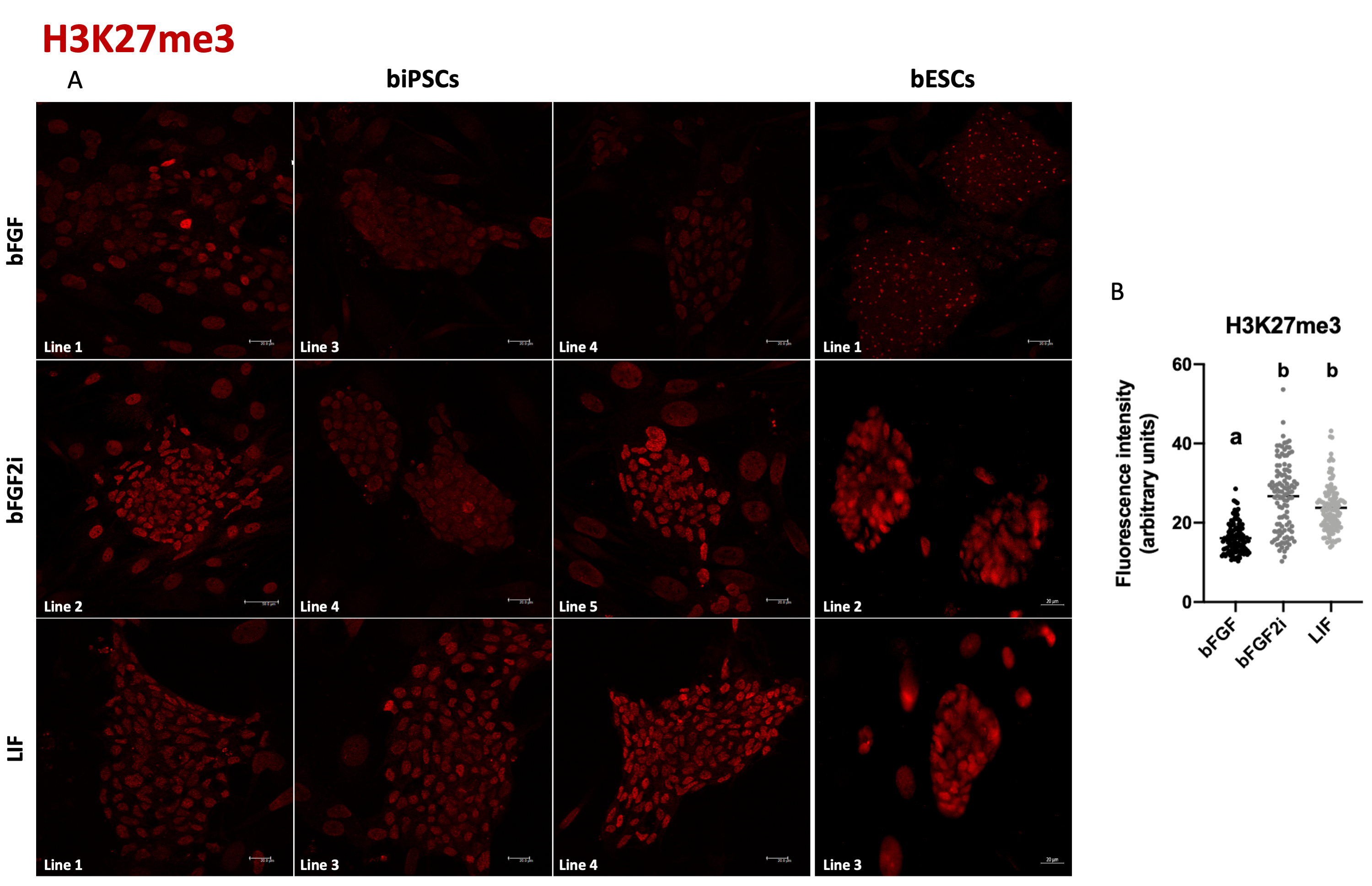

Supplement: Supplementary file 4 [file Image5.TIFF]

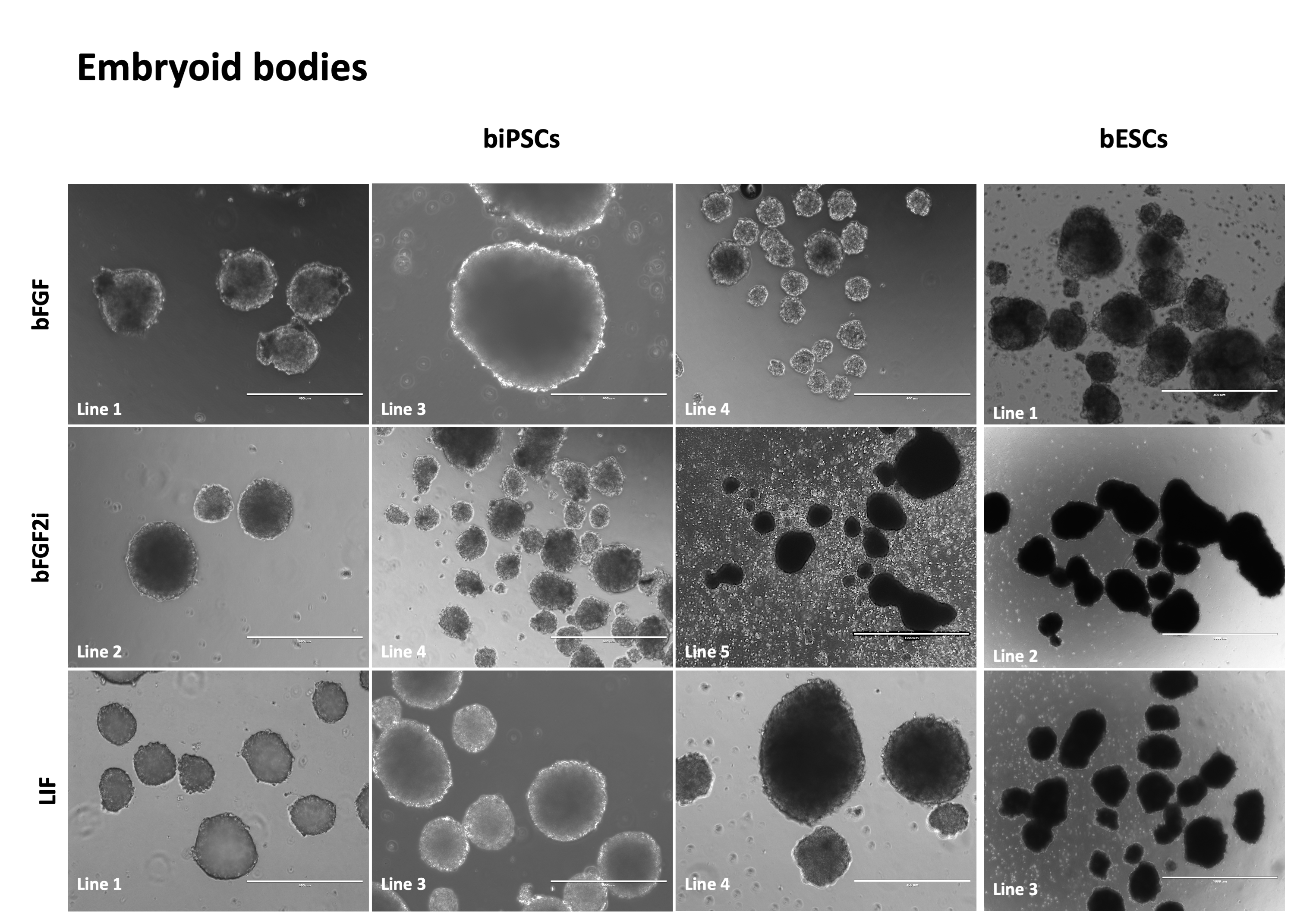

Supplement: Supplementary file 5 [file Image6.TIFF]

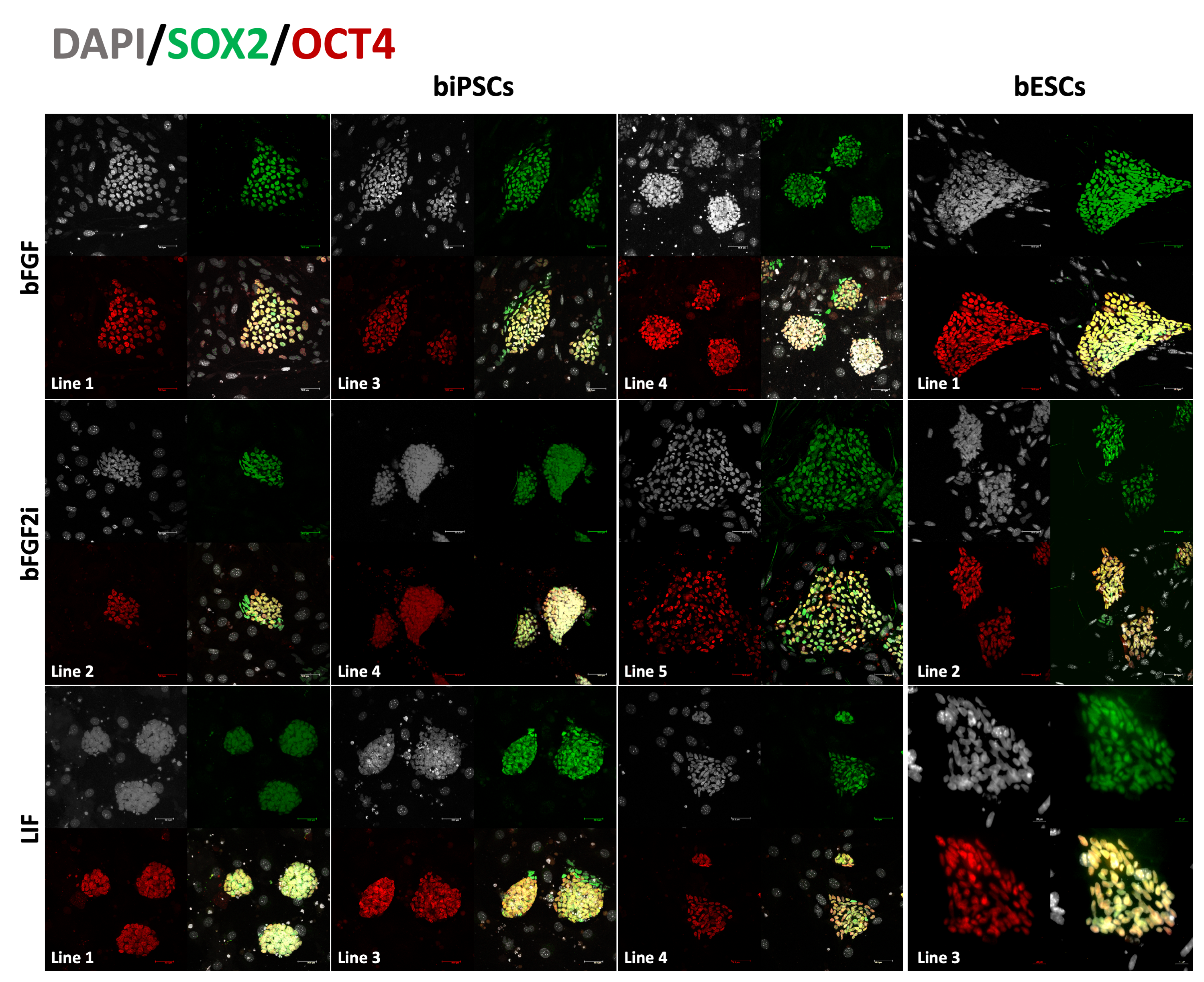

Supplement: Supplementary file 6 [file Image2.TIFF]

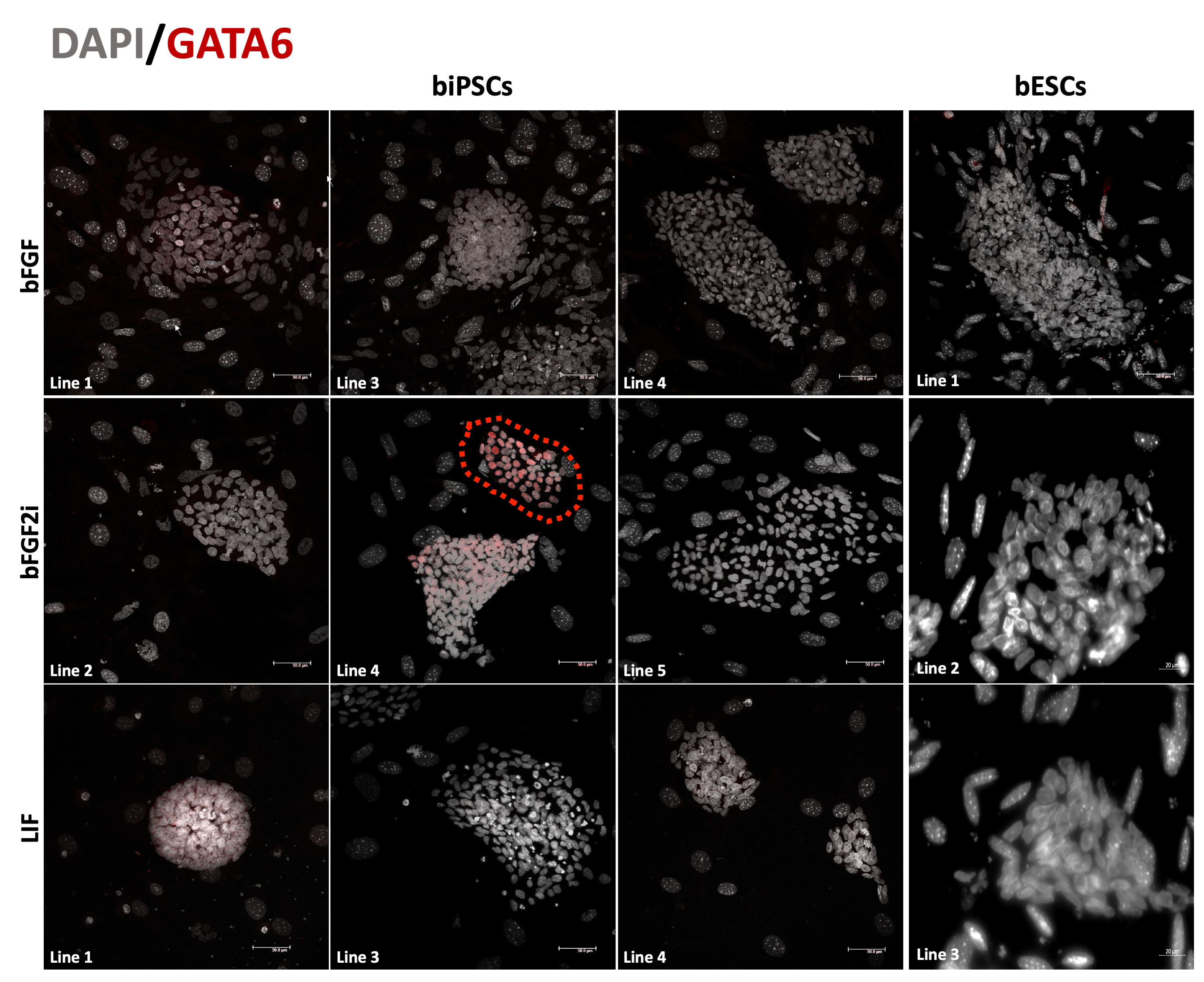

Supplement: Supplementary file 7 [file Image4.TIFF]
