# Supplementary material for: Acquisition and maintenance of pluripotency are influenced by fibroblast growth factor, leukemia inhibitory factor, and 2i in bovine-induced pluripotent stem cells
Source: Front Cell Dev Biol. 2022 Sep 14;10:938709. doi: 10.3389/fcell.2022.938709 (PMC9515551; doi:10.3389/fcell.2022.938709)
Supplement: Supplementary file 3 [file Table1.DOCX]

Supplementary Material

Legends of supplementary figures

Supplementary figure 1. Morphology of biPSCs and bESCs colonies.

Supplementary figure 2. Immunofluorescence to SOX2 and OCT4 in biPSCs and bESCs colonies.

Supplementary figure 3. Immunofluorescence to NANOG in biPSCs and bESCs colonies.

Supplementary figure 4. Immunofluorescence to GATA6 in biPSCs and bESCs colonies.

Supplementary figure 5. A, Immunofluorescence to H3K27me3 in biPSCs and bESCs colonies. B, Graphical representation of fluorescence intensity to H3K27me3 in bovine iPSCs. The box plot graphic represented the fluorescence intensity of each analyzed cell and the data was expressed as median ± interquartile interval.

Supplementary figure 6. Embryoid bodies (EBs) produced from biPSCs and bovine induced pluripotent stem cells..
